# Pregnancy Outcomes in a Cohort of Patients with Inflammatory Bowel Disease: Data from a Multidisciplinary Clinic in a Tertiary Center

**DOI:** 10.3390/jcm12124120

**Published:** 2023-06-18

**Authors:** Irit Avni Biron, Lior Hayat, Jacob E. Ollech, Hagar Banai-Eran, Bar Narkis, Ohad Houri, Maor H. Pauker, Vardit Shay, Iris Dotan, Eran Hadar, Henit Yanai

**Affiliations:** 1 Inflammatory Bowel Disease Center, Division of Gastroenterology, Rabin Medical Center, Petah-Tikva 4919001, Israel; 2 Sackler Faculty of Medicine, Tel Aviv University, Tel Aviv 6997801, Israel; 3 Helen Schneider Hospital for Women, Rabin Medical Center, Petah-Tikva 4941492, Israel

**Keywords:** pregnancy, inflammatory bowel disease, outcomes

## Abstract

Background: Inflammatory bowel disease (IBD) can have an impact on pregnancy outcomes due to the effect of the disease activity and medication use. This study aimed to evaluate the pregnancy outcomes in IBD patients treated at a multidisciplinary clinic. Methods: This study was a retrospective cohort study including consecutive pregnant patients with IBD having a singleton gestation attending a multidisciplinary clinic between 2012 and 2019. The IBD activity and management throughout gestation were assessed. The pregnancy outcomes included: adverse neonatal and maternal outcomes, mode of delivery, and three integrative outcomes: (1) a favorable pregnancy outcome, (2) a poor pregnancy outcome, and (3) an unfavorable maternal outcome. The IBD pregnant cohort was compared with a cohort of non-IBD pregnant women delivering at the same shift. Multivariable logistic regression was used for risk assessment. Results: Pregnant women with IBD (141) and without (1119) were included. Mean maternal age was 32 [±4] years. Patients with IBD had a higher rate of nulliparity (70/141 (50%) vs. 340/1119 (30%), *p* < 0.001) and lower BMI (21.42 kg/m^2^ (19.18–23.44) vs. 22.48 (20.31–25.59), *p* = 0.002). All the other characteristics were comparable. Most patients with IBD 124/141 (88%) were in clinical remission at conception; with maintenance therapy in 117/141 patients (83%). A third of the patients, 43/141 (30.5%), were treated with biologics. Exacerbation occurred during pregnancy in 51/141 (36%). The majority of the maternal and neonatal outcomes and all the composite outcomes were comparable between the patients with IBD and the women without IBD. Cesarean delivery was more frequent in patients with IBD (49/141 (34.8%) vs. 270/1119 (24.1%), *p* = 0.021). IBD was not associated with composite outcomes. Conclusions: In pregnant patients with IBD followed at a multidisciplinary clinic, the pregnancy outcomes were encouraging and comparable to those of the women without IBD.

## 1. Introduction

Inflammatory bowel disease (IBD), including Crohn’s disease (CD) and ulcerative colitis (UC), often affect women in their reproductive years [1,2].

IBD diagnosis raises concerns regarding the impact of disease activity and medication use on pregnancy outcomes [3,4]. Pregnant women with IBD are considered predisposed to adverse pregnancy outcomes compared to the general population [5,6,7]. In population cohort studies and meta-analyses, an IBD diagnosis and specifically active disease at conception and during pregnancy, were associated with adverse pregnancy outcomes [8,9,10,11,12,13,14,15], while in cohort studies from dedicated or multidisciplinary clinics, pregnancy outcomes were generally favorable [16,17,18,19]. This study aimed to assess the pregnancy outcomes of women with IBD treated at an IBD–Maternal-Fetal Medicine (MFM) clinic that provides multidisciplinary care for women with IBD during pregnancy. 

## 2. Materials and Methods

### 2.1. Study Setting

A dedicated, multidisciplinary IBD–MFM clinic was established in 2011 at Rabin Medical Center (RMC) in Israel, a major referral center for patients with IBD. Patients were referred to the clinic for pre-conception consultation or at early pregnancy, from the RMC IBD center, as well as from community centers in Israel. The clinic was routinely operated by an IBD expert and an MFM expert. Patients were co-evaluated conjointly at each visit. At the first encounter, patients were comprehensively assessed for IBD-related course, obstetrical history, and current status. During follow-up visits, in addition to clinical parameters, medication use, and adherence, vitamin supplementation, nutrition, and lifestyle factors were discussed and addressed. Patients were examined at least once at each pregnancy trimester, and more frequently as needed during disease exacerbations or if pregnancy complications arose. Recommendations regarding IBD and obstetrical management, including the mode of delivery, were provided. Additionally, consultations by a dedicated dietitian and a colorectal surgeon were provided as required. 

### 2.2. Study Design

In this retrospective cohort study, we reviewed the medical records of women with IBD with a singleton gestation, who were followed at our clinic throughout pregnancy, and delivered at RMC between 2012–2019. For women with more than one pregnancy, a single pregnancy was randomly selected. Exclusion criteria included multiple gestations, birth outside of RMC, and insufficient follow-up, precluding complete information on the disease, pregnancy course, and delivery outcomes. 

Outcomes of the women with IBD were compared with the outcomes of all the women without IBD, who delivered at the same hospital shift; this was chosen to adjust for possible variations in medical decisions that may have impacted the outcomes of interest [20,21]. Patient’s demographics, IBD-related data, obstetrical and neonatal characteristics, and outcomes were captured and recorded from the computerized medical records. 

### 2.3. Outcomes and Definitions

#### Pregnancy Outcomes

Multiple pregnancy and neonatal outcomes were collected, as specified in the outcomes table. In order to assess the comprehensive and meaningful outcomes for patients, we defined three specific integrated endpoints: (1) Favorable pregnancy outcomes: an integrated outcome including the term (≥37 + 0 gestational weeks), lack of small for gestational age (SGA), and 5 min Apgar score ≥8. (2) Poor pregnancy outcome: an integrated outcome including early preterm birth (<34 + 0 gestational weeks) and/or severe SGA (birthweight < 3%) and/or stillbirth. (3) Unfavorable maternal outcome: an integrated outcome including at least one of the following: post-partum hemorrhage, gestational diabetes mellitus, chorioamnionitis, gestational hypertension, preeclampsia, eclampsia, hemolysis, elevated liver enzymes low platelet syndrome, prolonged hospitalization, or unplanned cesarean delivery.

### 2.4. IBD Activity

Disease activity was assessed clinically and categorized into three levels at each pregnancy trimester according to the following parameters: (1) No clinical activity: lack of IBD-related symptoms, stable medical therapy, no use of corticosteroids, and no IBD-related hospitalization or surgery; (2) Mild–moderate clinical activity: requirement for optimization of 5-Aminosalicylates therapy or addition of topical 5-Aminosalicylates or topical corticosteroid therapy, no use of systemic corticosteroids or IBD-related hospitalization or surgery; (3) Severe activity: requirement for systemic corticosteroids, treatment escalation, or initiation of new biologic therapy, IBD-related hospitalization or surgery, or further evaluation with Magnetic Resonance Enterography or endoscopy. IBD exacerbation was defined as escalation of activity level compared to baseline. 

### 2.5. Statistical Analysis

Categorical variables were presented as frequency and percentage, while continuous variables were summarized as the median and interquartile range (IQR) or mean and standard deviation (SD). Comparison of categorical variables was performed using the Chi-square test or Fisher’s exact test, and continuous variables were compared using the Student’s independent T-test or Mann–Whitney U test as appropriate. The Bonferroni method was used to adjust for multiple comparisons. Multivariable logistic regression using a backward stepwise method for variable selection (*p*-value > 0.1 on the Wald test) was used for variable removal to assess the risk for all three integrated outcomes. Risk was adjusted for maternal age, smoking status, parity, mode of conception, chronic hypertension, diabetes mellitus (type 1, type 2), previous abortion, and previous cesarean delivery. Risk was reported as adjusted odds ratio (adj OR) and 95% confidence interval (CI). *p*-values < 0.05 were considered significant. Analysis was performed using IBM SPSS statistics, version 28.0, IBM Corp., Armonk, NY, USA, 2021.

## 3. Results

### 3.1. Patients and Baseline Characteristics

Overall, 141/222 patients with IBD were eligible for inclusion. This cohort was compared with a cohort of 1119 women without IBD (Supplementary Figure S1—study population disposition). The mean maternal age was 32 (±4) years in both cohorts. The patients with IBD had a significantly lower BMI at conception (21.42 vs. 22.48, *p* = 0.002) and a higher rate of nulliparity (70/141 (50%) vs. 340/1119 (30%) *p* = 0.001). Hypothyroidism was noted at a higher rate in patients with IBD (14/141 (9.9%) vs. 60/1119 (5.4%), *p* = 0.036). Rates of smoking, mode of conception, history of an abortion, cesarean delivery, and comorbidities including diabetes mellitus and chronic hypertension were comparable between groups (Supplementary Table S1).

### 3.2. IBD Characteristics and Management 

There was diagnosis of CD in 85/141 (60%) and UC in 56/141 (40%). The median disease duration was 4 (1–10) years. Most patients, 117/141 (83%), received maintenance therapy during pregnancy; biologic agent 43/141 (30.5%), thiopurines 30/141 (21.3%), combination-therapy 9/141 (6.3%); 24/141 (17%) were not receiving IBD-related medications prior to the pregnancy and at conception, Table 1. 

In total, 124/141 (88%) patients were in clinical remission at conception. An exacerbation occurred during pregnancy in 51/141 (36%) patients. Mild–moderate exacerbation was observed in 27/141 (19%) patients and severe exacerbation was observed in 24/141 (17%) patients. Exacerbations were significantly more frequent among patients with UC as compared to CD (27/56 (48.2%) vs. 24/85 (28.2%), *p* = 0.015); see Table 2 for disease activity per trimester stratified by the disease type. Patients were treated by the IBD–MFM team according to the guidelines, see Supplementary Table S2.

Of note, in 28/141 (19.9%) of the patients, who were all in clinical remission, a shared decision was made to interrupt the biologic treatment at the third trimester, with the intent to reduce fetal drug exposure at late pregnancy, with the median last dose administered at week 30 (27–33) weeks; remission was maintained uneventfully in 25/28 (89%) patients.

### 3.3. Pregnancy Outcomes

Overall, all the obstetrical and neonatal outcomes were satisfactory for the entire cohort; see Table 3 for the detailed outcomes for the IBD and non-IBD cohorts. The median gestational age at birth and the birth weight were lower for neonates delivered by the patients with IBD compared with the women without IBD (38 [(38–39) vs. 39 (38–40) gestational weeks, *p* = 0.003) and (3148 (2881–3385) vs. 3220 g (2928–3492), *p* = 0.039), respectively.

No significant differences were demonstrated in the rates of preterm delivery (12/141 (8.5%) vs. 96/1119 (8.6%), *p* = 0.999) and SGA (10/141 (7.1%) vs. 50/1119 (4.5%), *p* = 0.203). The rates of stillbirth were low, and comparable between groups (0/141 (0%) vs. 9/1119 (0.8%), *p* = 0.609). The cesarean deliveries were conducted at a higher proportion in the patients with IBD as compared to the women without IBD (49/141, (34.8%), vs. 270/1119 (24.1%), *p* = 0.021). The rates of planned and unplanned cesarean were comparable between the cohorts (35/49 (71.4%) vs. 174/270 (64.4%), *p* = 0.415 and 14/49 (28.6%) vs. 96/270 (35.6%), *p* = 0.415, respectively). Cesarean delivery was conducted in a comparable rate in the patients with CD and UC (32/85 (37.6%) vs. 17/56 (30%), *p* = 0.347). The most common indication for a planned cesarean delivery among the patients with CD was active perianal disease, followed by a previous cesarean delivery (12/22 (54.54%) and 9/22 (40.90%) patients, respectively). The most common indication of planned cesarean delivery among the patients with UC and the women without IBD was a previous cesarean delivery (8/12 (66.66%) and 106/174 (60.91%) patients, respectively). Favorable pregnancy outcome was achieved by 119/141 (84.4%) patients with IBD; these rates were similar among women without IBD (976/1119 (87.2%)), *p* = 0.354. Poor pregnancy outcome, and unfavorable maternal outcome, were demonstrated in 5/141 (3.5%) and 40/141 (28.4%) of the patients with IBD, respectively, and these rates were comparable for women without IBD.

### 3.4. Risk Factors for Adverse Pregnancy Outcomes

**Figure 1:**
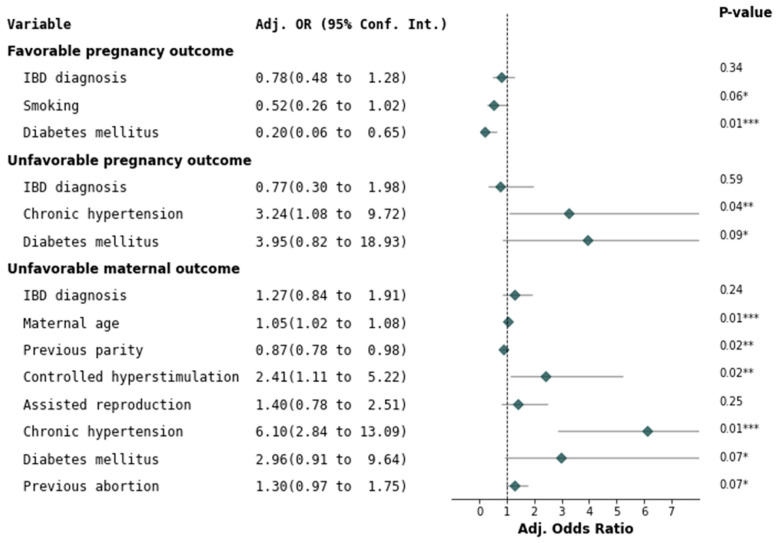
Risk factors for integrated outcomes. (Diabetes mellitus—type 1, type 2). * *p*-value < 0.1, ** *p* value < 0.05, *** *p* value ≤ 0.01.

Pre-term birth occurred at a higher rate in the patients having severe exacerbation during pregnancy as compared to the patients with mild–moderate exacerbation and quiescent disease (25% (6/24) vs. 7.4% (2/27) and 4.4% (4/90), *p* = 0.006). Adjusting for multiple comparison (Bonferroni correction) revealed that the severe active disease was significantly different from all other groups (*p* = 0.006), Supplementary Table S4. Based on the multivariable logistic regression analysis, a diagnosis of IBD was not associated with either a favorable or a poor pregnancy outcome, nor an unfavorable maternal outcome (Figure 1).

## 4. Discussion

In this cohort study of patients with IBD followed at a multidisciplinary IBD–MFM clinic, the obstetrical and neonatal outcomes were favorable, and comparable to those of women without IBD. The integrated outcomes were also comparable, and a diagnosis of IBD was not associated with either a favorable, or a poor pregnancy outcome, nor an unfavorable maternal outcome. Most of the patients with IBD in this cohort conceived while in clinical remission, most continued their maintenance therapy during pregnancy, and 36.2% exacerbated during pregnancy. In the patients with severe activity during pregnancy, a higher rate of pre-term delivery was observed. Disease exacerbation was more prevalent among the patients with UC compared with CD (48.2% vs. 28.2%, *p* = 0.015), and cesarean deliveries were more common in the patients with IBD compared to the women without IBD (34.8% vs. 24.1%, *p* = 0.021).

Multiple nationwide population studies have demonstrated that patients with IBD have worse pregnancy outcomes when compared to the general population, mostly when the disease is active during conception and pregnancy [8,9,10,11,15]. Therefore, the current guidelines support conception at times of remission and continuation of the IBD medications during pregnancy, in conjunction with prompt identification and management of exacerbations by a multidisciplinary team [5]. This study supports the current therapeutic paradigm, demonstrating that in patients conceiving while in clinical remission (87.1% and 89.3% of patients with CD and UC), and receiving dedicated supervision during pregnancy, obstetrical and neonatal outcomes were satisfactory. In congruency, the patients who had severe disease activity during pregnancy, had higher rates of pre-term births (severe—25%, mild–moderate—7.4%, quiescent disease—4.4%), supporting the need for dedicated follow-up and care.

In agreement with our findings, the data from other specialized clinics that provide pre-conception counseling and IBD care during pregnancy have also demonstrated overall favorable pregnancy outcomes [16,17,18] (84.4%) with exacerbations affecting one-third of patients; in Rotterdam, the exacerbation rate was 29.7% and, in both Rotterdam and Leeds, active disease during pregnancy was not associated with adverse outcomes [17,19].

The patients with UC in our cohort had more frequent exacerbations compared with the patients with CD (48.2% vs. 28.2%, *p* = 0.015). In fact, previous data indirectly suggest that pregnant patients with UC are prone to exacerbations during pregnancy; data from the Shaare Zedek Medical Center and the Rotterdam Clinic demonstrated that among woman with quiescent IBD there was a higher risk for exacerbation among women with UC compared to CD [17,22], and the EpiCom cohort study showed that pregnant women with UC were at risk for exacerbation compared to non-pregnant women with UC (RR 2.19; 95% CI: 1.25–3.97, *p* = 0.004), but it was not shown for CD [23].

Higher rates of cesarean deliveries in patients with IBD (mostly CD) have been previously reported [24]. Similarly, in our study, a higher rate of cesarean delivery was noted in the IBD compared to non-IBD pregnancies (34.8% vs. 24.1%, *p* = 0.021). In contrast to previous publications demonstrating a higher rate of planned cesarean section in the IBD compared to non-IBD population [18,19], the rates of planned cesarean section in our study were comparable. This difference was probably related to a higher rate of multiparous women in the non-IBD group, to a higher birth-rate in Israel as compared to Europe, and to the preference of a cesarean delivery over vaginal in following labors after a first cesarean. Indeed, the most frequent indication for planned cesarean delivery differed between groups with active perianal disease in the IBD group, and previous cesarean delivery in the non-IBD.

Our study had several strengths: multidisciplinary care was given to all the patients throughout pregnancy by the same dedicated team, contributing to a unified approach. With the intent of providing patients and caregivers with more approachable data regarding pregnancy outcomes in women with IBD, the integrated outcomes that are of interest to the patients’ population were used here for the first time, demonstrating that favorable pregnancy outcomes (84.4%) were comparable to women without IBD (87.2%) and that the IBD diagnosis per se was not associated with poor pregnancy outcomes.

Nevertheless, this study was not without limitations: as most patients conceived while in clinical remission, the entire spectrum of IBD activity at conception was not demonstrated and therefore the results cannot be generalized. The study population was relatively small, and severe adverse pregnancy outcomes were relatively rare; therefore, the interpretation of the results should be taken with caution; this issue was addressed by the generation of integrated outcomes. Clinical activity questionnaires were not used to assess the clinical activity, and fecal calprotectin was not routinely used; therefore, the assessment of the clinical activity was mostly subjective. However, the clinical activity questionnaires have not been validated in the pregnancy population and they are not systematically used in daily clinical practice; therefore, the study results better represent the “real-world” practice. Additionally, during the study period, fecal calprotectin was not routinely used in Israel. Even with the improved availability nowadays, this test is not routinely provided per trimester and is not embraced by all patients. This study did not provide data regarding congenital malformations, spontaneous abortions, and neonatal intensive care admissions due to the structure of the data registration at our institute, and the lack of informed consent to retrieve more detailed neonatal data. However, this study’s aim was not to describe the medication safety, but to give a more generalized overview of the pregnancy outcomes in the patients with IBD treated at a dedicated clinic.

In conclusion, our findings demonstrated that in patients conceiving while in clinical remission, and receiving dedicated supervision during pregnancy, the pregnancy outcomes were satisfactory, and were comparable to those of the general population. These outcomes may add to the discussion between caregivers and patients, delivering an encouraging message regarding the importance of adherence to the current guidelines. Finally, the results support multidisciplinary care for pregnant patients with IBD, such as that provided in our IBD–MFM clinic.

## Figures and Tables

**Table 1:** Baseline IBD characteristics and medication history.

	All IBD	Crohn’s Disease	Ulcerative Colitis	*p*-Value
	n = 141	n = 85	n = 56	
Maternal age, years, mean ± SD	32 ± 4	32 ± 4	31 ± 5	0.816
Median age at IBD diagnosis	24 (19.25–28)	24 (19–27)	25 (21–29)	0.147
Disease duration, years	4 (1–10)	4 (1–12)	3 (0–8)	0.018
Baseline Body Mass Index, kg/m^2^, Median [IQR]	21.42 [19.18–23.44]	21.52 [19.21–23.44]	21.265 [19.48–23.185]	0.971
Smoking, n (%)	10 (7.1)	8 (9.4)	2 (3.6)	0.315
EIM, n (%)	38 (26.9)	27 (31.2)	11 (7.8)	0.028
Montreal A, n (%), A1/A2/A3		21 (24.7)/63 (74)/1 (1.3)		
Montreal L, n (%), L1/L2/L3/L4		34 (40)/6 (7.1)/41 (48.2)/4 (4.7)		
Montreal B, n (%), B1/B2/B3		63 (74)/13 (15.3)/9 (10.6)		
Perianal fistulizing disease, n (%)		13 (15.29)		
Proctitis/Left sided colitis/Pancolitis, n (%)			18 (34)/19 (36)/16 (30)	
History of IBD related intestinal resection, n (%)	18 (12.8)	15 (17.6)	3 (5.3)	0.039
**Previous IBD medications, n (%)**				
Mesalamine	90 (63.8)	47 (55.3)	43 (76.8)	0.012
Sulfasalazine	8 (5.7)	4 (4.7)	4 (7.1)	0.713
Thiopurines	67 (47.5)	50 (58.8)	17 (30.3)	0.001
Methotrexate	5 (3.5)	4 (4.7)	1 (1.7)	0.648
Infliximab	33(23.4)	22 (25.9)	11 (19.6)	0.424
Adalimumab	29(20.6)	24 (28.2)	5 (8.9)	0.006
Golimumab	1 (0.7)	0 (0)	1 (1.7)	0.397
Vedolizumab	2 (1.4)	1 (1.2)	1 (1.7)	0.999

**Table 2:** IBD activity at conception and throughout pregnancy.

n, (%)	IBD n = 141			Crohn’s Disease n = 85			Ulcerative Coslitis n = 56			*p*-Value
	Quiescent	Mild-Moderate	Severe	Quiescent	Mild- Moderate	Severe	Quiescent	Mild-Moderate	Severe	
Preconception	124 (88)	14 (10)	3 (2)	74 (87.1)	8 (9.4)	3 (3.5)	50 (89.3)	6 (10.7)	0 (0)	0.808
1st trimester	115 (81.5)	19 (13.6)	7 (4.9)	72 (84.7)	7 (8.2)	6 (7)	43 (76.8)	12 (21.4)	1 (1.8)	0.038
2nd trimester	104 (73.8)	21 (14.9)	16 (11.3)	64 (75.3)	13 (15.3)	8 (9.4)	40 (71.4)	8 (14.3)	8 (14.3)	0.798
3rd trimester	101 (71.6)	24 (17)	16 (11.3)	65 (76.5)	13 (15.3)	7 (8.2)	36 (64.3)	11 (19.6)	9 (16.1)	0.234
Throughout pregnancy	90 (64)	27 (19)	24 (17)	61 (71.8)	11 (12.9)	13 (15.3)	29 (51.8)	16 (28.6)	11 (19.6)	0.033

**Table 3:** Obstetric, neonatal, and integrated outcomes in the IBD and non-IBD cohorts.

	Total N = 1260	IBD n = 141	Non-IBD n = 1119	*p*-Value
**Obstetric outcomes**				
Gestational diabetes, n (%)	125 (10)	19 (13.5)	106 (9.6)	0.178
Gestational hypertension, n (%)	11 (0.9)	2 (1.4)	9 (0.8)	0.353
Preeclampsia, n (%)	21 (1.6)	3 (2.1)	18 (1.4)	0.65
Preeclampsia without severe features, n (%)	8 (0.6)	2 (1.4)	6 (0.5)	0.805
Preeclampsia with severe features, n (%)	5 (0.4)	0 (0)	5 (0.4)	
HELLP syndrome, n (%)	8 (0.6)	1 (0.7)	7 (0.6)	
Eclampsia, n (%)	2 (0.1)	1 (0.7)	1 (0.08)	0.081
Placental abruption, n (%)	14 (1.1)	2 (1.4)	12 (1.1)	0.664
Placenta previa, n (%)	19 (1.5)	18 (1.6)	1 (0.7)	0.713
Chorioamnionitis, n (%)	4 (0.3)	1 (0.7)	3 (0.3)	0.378
Premature preterm rupture of membranes, n (%)	72 (5.7)	26 (18.4)	46 (4.1)	<0.001
Postpartum hemorrhage, n (%)	22 (1.7)	8 (5.7)	14 (1.3)	0.002
Mode of delivery				
Vaginal delivery, n (%)	841 (66.7)	81 (57.4)	760 (67.9)	0.021
Assisted delivery, n (%)	100 (7.9)	11 (7.8)	89 (8)	
Cesarean delivery, n (%)	319 (25.3)	49 (34.8)	270 (24.1)	
Cesarean delivery type				
Planned Cesarean, n (%)	209 (65.5)	35 (71.4)	174 (64.4)	0.415
Unplanned Cesarean, n (%)	110 (34.5)	14 (28.6)	96 (35.6)	
Maternal hospitalizations, n (%)	231 (18.3)	39 (27.7)	192 (17.1)	0.011
IBD indication, n (%)		12 (8.5)		
Obstetrical indication, n (%)		27 (19.1)		
**Neonatal outcomes**				
Gestational age at delivery, week, median, (IQR)	39 (38–40)	38 (38–39)	39 (38–40)	0.003
Preterm delivery, n (%)	108 (8.6)	12 (8.5)	96 (8.6)	0.999
Early preterm delivery, n (%)	34 (2.7)	3 (2.1)	31 (2.8)	0.999
Stillbirth, n (%)	9 (0.7)	0 (0)	9 (0.8)	0.609
Birth weight (grams), median, (IQR)	3206 (2922–3487)	3148 (2881–3385)	3220 (2928–3492)	0.039
Low birth weight <2500 gr, n (%)	115 (9.1)	14 (9.9)	101 (9)	0.756
Small for gestational age, n (%)	60 (4.8)	10 (7.1)	50 (4.5)	0.203
Severe Small for gestational age, n (%)	16 (1.2)	1 (0.7)	15 (1.3)	0.528
5-Min Apgar score, mean ± SD	9.8 ± 1.04	9.9 ± 0.539	9.8 ± 1.09	0.244
5-Min Apgar <7, n (%)	19 (1.5)	1 (0.7)	18 (1.6)	0.713
**Integrated outcomes**				
Favorable pregnancy outcome, n (%)	1095 (86.9)	119 (84.4)	976 (87.2)	0.354
Poor pregnancy outcome, n (%)	56 (4.4)	5 (3.5)	51 (4.6)	0.672
Unfavorable maternal outcome, n (%)	288 (22.9)	40 (28.4)	248 (22.2)	0.111

Preterm delivery: <37 weeks, Early preterm delivery: <34 weeks, Small for gestational age: <10th percentile birth weight, Severe Small for gestational age: <3rd percentile birth weight, Favorable pregnancy outcome: term birth ≥ 37 weeks, appropriate for gestational age and Apgar ≥ 8, Poor pregnancy outcome: early preterm birth < 34 weeks and/or severe small for gestational age < 3% and/or Apgar ≤ 6 and/or stillbirth, Unfavorable maternal outcome: any of the following: postpartum hemorrhage (PPH), gestational diabetes mellitus (GDM), chorioamnionitis, gestational hypertension, preeclampsia with and without severe features, eclampsia, HELLP syndrome, prolonged hospitalization or unplanned cesarean delivery.

## Data Availability

Data cannot be shared for ethical/privacy reasons. The data underlying this article cannot be shared publicly due to the privacy of the individuals that participated in the study. The data will be shared on reasonable request with the corresponding author.

## Supplementary Materials

The following supporting information can be downloaded at: https://www.mdpi.com/article/10.3390/jcm12124120/s1, Figure S1: Study population—disposition; Table S1: Baseline demographics and characteristics; Table S2: Drug therapy during pregnancy; Table S3: Management of disease exacerbations; Table S4: Major outcomes stratified by IBD disease activity throughout pregnancy.
