# Supplementary material for: Pregnancy Outcomes in a Cohort of Patients with Inflammatory Bowel Disease: Data from a Multidisciplinary Clinic in a Tertiary Center
Source: J Clin Med. 2023 Jun 18;12(12):4120. doi: 10.3390/jcm12124120 (PMC10299482; doi:10.3390/jcm12124120)
Supplement: Supplementary file 1 [file jcm-12-04120-s001.zip › jcm-2398232-supplementary.pdf]

**Figure S1:** Study population disposition

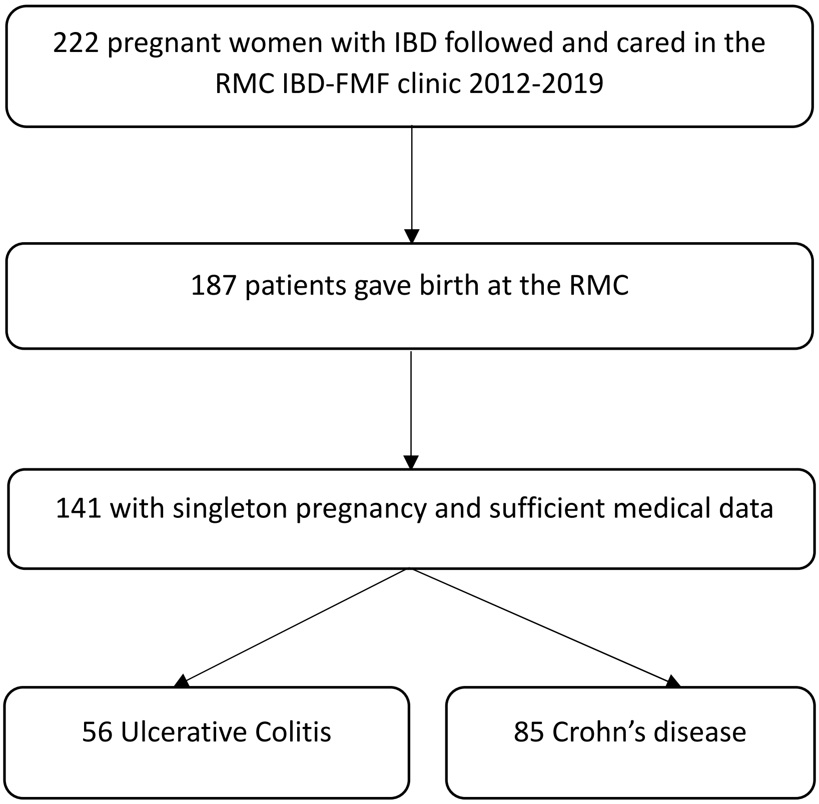

Table S1: Baseline demographics and characteristics

| | IBD n=141 | Non-IBD n=1119 | P value |
|---|---|---|---|
| Maternal age, years, mean ± SD | 32±4 | 32±5 | 0.935 |
| Pre-pregnancy Body Mass Index, Kg/m$^2$, Median [IQR] | 21.42 [19.18-23.44] | 22.48 [20.31-25.59] | 0.002 |
| Smoking, n (%) | 10 (7.1) | 44 (3.9) | 0.117 |
| Mode of Conception | | | 0.228 |
| Controlled Ovarian Hyperstimulation, n (%) | 6 (4.3) | 23 (2.1) | |
| Assisted Reproduction, n (%) | 8 (5.7) | 53 (4.7) | |
| Nulliparity, n (%) | 70 (50) | 340 (30) | 0.001 |
| Previous abortion, n (%) | 48 (34) | 372 (33) | 0.88 |
| Previous cesarean delivery, n (%) | 24 (17) | 192 (17) | 0.957 |
| Chronic Hypertension, n (%) | 5 (3.5) | 26 (2.3) | 0.382 |
| Type 1 or 2 Diabetes mellitus, n (%) | 0 (0) | 12 (1) | 0.999 |
| Hypothyroidism, n (%) | 14 (9.9) | 60 (5.4) | 0.036 |
| Birth shift | | | 0.714 |
| Morning (7:00- 15:00), n (%) | 60 (42.6) | 502 (44.9) | |
| Evening (15:00-23:00), n (%) | 42 (29.8) | 343 (30.7) | |
| Night (23:00-7:00), n (%) | 39 (27.7) | 274 (24.5) | |

Table S2: Drug therapy during pregnancy

| | IBD n=141 | Crohn's Disease n=85 | Ulcerative colitis n=56 | P-value |
|---|---|---|---|---|
| Drug therapy during pregnancy, n (%) | 117 (83) | 65 (76.5) | 52 (93) | 0.012 |
| Mesalamine | 55 (39) | 19 (22.4) | 36 (64) | <0.001 |
| Topical mesalamine | 27 (19.1) | 2 (2.4) | 25 (44.6) | <0.001 |
| Thiopurine | 30 (21.3) | 24 (28.2) | 6 (10.7) | 0.02 |
| Biology treatment during pregnancy, n (%) | 43 (30.5) | 30 (35.3) | 13 (23.2) | 0.127 |
| Anti TNF combo | 8 (5.7) | 6 (7.1) | 2 (3.6) | 0.478 |
| Anti TNF mono | 41 (29.1) | 29 (34.2) | 12 (21.4) | 0.13 |
| infliximab | 15(10.6) | 8 (9.4) | 7 (12.5) | 0.586 |
| adalimumab | 25 (17.7) | 20 (23.6) | 5 (8.9) | 0.041 |
| certolizumab | 1(0.7) | 1 (1.2) | 0 (0) | 0.999 |
| vedolizumab | 2(1.4) | 1 (1.2) | 1 (1.7) | 0.999 |
| Third trimester biologic discontinuation | 28(19.9) | 19 (22.4) | 9 (16.1) | 0.999 |
| Week of last biologic | 30 [27-33] | 31 [23-32] | 28 [28-33] | 0.239 |

Table S3: Management of disease exacerbations

| n, (%) | IBD<br>n=141 | Crohn's Disease<br>n=85 | Ulcerative colitis<br>n=56 | P-value |
|---|---|---|---|---|
| Budesonide | 5 (3.5) | 2 (2.0) | 3 (5.3) | 0.386 |
| Prednisone | 20 (14.2) | 11 (12.9) | 9 (16.1) | 0.628 |
| Optimization of biologic | 6 (4.3) | 3 (3.5) | 3 (5.3) | 0.682 |
| Initiation of Anti-TNF | 3 (2.1) | 2 (2.3) | 1 (1.7) | 0.999 |
| optimization of oral 5-ASA | 19 (13.4) | 3 (3.5) | 16 (28.5) | 0 |
| optimization / initiation of rectal 5-ASA | 17 (12) | 2 (2.3) | 15 (26.8) | 0 |
| Endoscopy during pregnancy | 9 (6.4) | 2 (2.3) | 7 (12.5) | 0.029 |
| IBD related hospitalization during pregnancy | 12 (85.1) | 6 (7.1) | 6 (10.7) | 0.541 |

Table S4: Major outcomes stratified by IBD activity throughout pregnancy

| n (%) | Quiescent<br>n=90 | Mild-moderate<br>n=27 | Severe<br>n=24 | P-value |
|---|---|---|---|---|
| Low birth weight <2500 gr | 6 (6.7) | 3 (11) | 5 (21) | 0.116 |
| Preterm delivery <37 weeks | 4 (4.4) | 2 (7.4) | 6 (25) * | 0.006 |
| Small for gestational age (<10th percentile birth weight) | 6 (6.7) | 2 (7.4) | 2 (8.3) | 0.085 |
| Gestational diabetes | 8 (8.9) | 3 (11) | 5 (21) | 0.505 |
| Preeclampsia toxemia (PET) | 3 (3.3) | 1 (3.7) | 0 (0) | 0.652 |
| Premature preterm rupture of membranes | 15 (16.7) | 4 (14.8) | 7 (29.2) | 0.323 |
| Postpartum hemorrhage | 5 (5.5) | 1 (3.7) | 2 (8.3) | 0.773 |
| Cesarean delivery | 34 (37.8) | 8 (33.3) | 7 (29.2) | 0.605 |
| Unplanned Cesarean | 10 (11.1) | 1 (3.7) | 3 (12.5) | 0.584 |
| Favorable pregnancy outcome | 79 (88) | 23 (85) | 17 (70) | 0.126 |
| Poor pregnancy outcome | 3 (3.3) | 1 (3.7) | 1 (4.2) | 0.979 |
| Unfavorable maternal outcome | 26 (29) | 4 (15) | 10 (42) | 0.103 |
